# Insights on the dissolution of water in an albite melt at high pressures and temperatures from a direct structural analysis

**DOI:** 10.1038/s41598-023-31043-7

**Published:** 2023-03-10

**Authors:** Robert A. Mayanovic, Alan J. Anderson, Devon Romine, Chris J. Benmore

**Affiliations:** 1 Department of Physics, Astronomy and Material Science, Missouri State University, Springfield, MO 65897 USA; 2 Department of Earth Sciences, St. Francis Xavier University, Antigonish, NS B2G 2W5 Canada; 3 X-Ray Science Division, Advanced Photon Source, Argonne National Laboratory, Argonne, IL 60439 USA

**Keywords:** Solid Earth sciences, Materials science

## Abstract

The water dissolution mechanism in silicate melts under high pressures is not well understood. Here we present the first direct structure investigation of a water-saturated albite melt to monitor the interactions between water and the network structure of silicate melt at the molecular level. In situ high-energy X-ray diffraction was carried out on the NaAlSi_3_O_8_-H_2_O system at 800 °C and 300 MPa, at the Advanced Photon Source synchrotron facility. The analysis of the X-ray diffraction data was augmented with classical Molecular Dynamics simulations of a hydrous albite melt, incorporating accurate water-based interactions. The results show that metal–oxygen bond breaking at the bridging sites occurs overwhelmingly at the Si site upon reaction with H_2_O, with subsequent Si–OH bond formation and negligible Al–OH formation. Furthermore, we see no evidence for the dissociation of the Al^3+^ ion from the network structure upon breaking of the Si–O bond in the hydrous albite melt. The results also indicate that the Na^+^ ion is an active participant in the modifications of the silicate network structure of the albite melt upon water dissolution at high P–T conditions. We do not find evidence for the Na^+^ ion dissociating from the network structure upon depolymerization and subsequent formation of NaOH complexes. Instead, our results show that the Na^+^ ion persists as a structure modifier with a shift away from Na–BO bonding to an increase in the extent of Na-NBO bonding, in parallel with pronounced depolymerization of the network. Our MD simulations show that the Si–O and Al–O bond lengths are expanded by about 6% in the hydrous albite melt compared to those of the dry melt at high P–T conditions. The changes in the network silicate structure of a hydrous albite melt at high pressure and temperature, as revealed in this study, must be considered in the advancement of water dissolution models of hydrous granitic (or alkali aluminosilicate) melts.

## Introduction

Water plays a key role in the dynamics of the Earth’s lithosphere. Developing a full model of the dissolution mechanism of water in silicate melts at the molecular level is critical for our understanding of geological processes such as the ascent, emplacement, and internal evolution of silicic magmas. The impact of water dissolution on the physical properties of silicate melts has been well documented. As the concentration of H_2_O in the melt increases, viscosity and nucleation rates decrease, while the electrical conductivity, compressibility, and the rate of diffusion of melt components increase^1–4^. The solubility of water in silicate melts has been shown to be pressure, temperature and composition dependent^5–8^. Bureau and Keppler showed that there is complete miscibility between H_2_O and silicate melts under upper mantle conditions^9^. The solubility of water is particularly variable in aluminosilicate melts, depending upon melt composition^10,11^. Most of the previous studies on the mechanistic aspect of the incorporation of water in silicate melts employed spectroscopic techniques (NMR, IR, Raman) of quenched hydrous glasses^10,12,13^. The primary issue with this approach is that the nature of incorporation of H_2_O and OH^–^ in the network structure of the melt is modified during quenching to a silicate glass^13,14^. Because water solubility is temperature dependent, quenched glasses retain H_2_O_m_/OH ratios (where H_2_O_m_ represents molecular water) at the glass transition temperature whereas these ratios are shown to decrease substantially under elevated temperature conditions in silicate melts at a given total water concentration. This was first shown from in situ IR measurements made on hydrous silicate melts at up to high P–T conditions by Nowak and Behrens^6^ and Shen and Kepler^15^, and most recently by Chertkova and Yamashita^16^.

In situ Raman and IR studies have provided insights into the structural aspects of how H_2_O and OH^–^ are incorporated at the molecular level in silicate melts^10^. A particularly notable work for its relevance to this study, made on aluminosilicate melt + H_2_O systems using Raman and FTIR, is by Mysen^17^. Although such studies are relevant to the interrogation of structure, they are indirect in nature and, as discussed in more detail below, the band assignments may occasionally be incorrect. Earlier studies on the solubility, compressibility, viscosity, and other studies, led to the development of the water-magma system thermodynamic model by Burnham^18^. Subsequent thermodynamic models of water dissolution in melts have improved upon the Burnham model^19,20^. The current consensus however is that these models are inaccurate primarily because the correct molecular level information of how water is incorporated into melts is lacking^13^. This can be directly attributed to our lack of a full understanding of the water dissolution mechanism and how this governs the structural modifications upon incorporation of water in silicate melts. The more sophisticated models which need to supplant the existing models require substantially more results from in situ structural experiments of water-melt systems at high P–T conditions.

The high P–T albite-water system is an ideal analogue for the study of hydrous silicic magmas^21–23^. Indirect structural data on hydrous albite glasses and melts have been obtained from NMR, Raman and IR measurements^10,13^. These studies generally agree that depolymerization of the network structure occurs in albite melts but disagree on the inferred nature of H_2_O–T–O–T reactions, where T indicates Si or Al, the precise role of the Al^3+^ and alkali ions within the modifications of the intermediate structure, and extent of depolymerization upon water incorporation. In this work, we present structural results from in situ high-energy X-ray diffraction (XRD) measurements made on the albite–water system at 800 °C and ~ 300 MPa. The structural analysis of the XRD data was assisted by Molecular Dynamics (MD) simulations that accurately account for the water-based chemical reactions with albite. By utilization of this combined approach, we can for the first time provide direct insights on how the intermediate structure of a silicate melt is affected by interaction with water at high P–T conditions.

**Figure 1:**
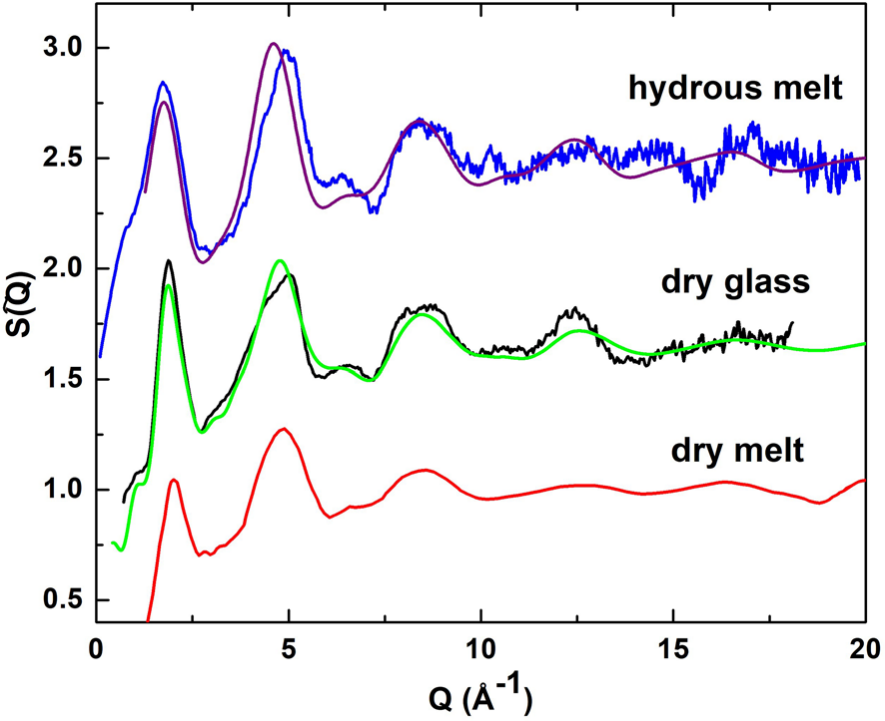
The experimental X-ray S(Q) structure factors for the dry albite glass (black line) and for the hydrous albite melt at 800 °C and approximately 300 MPa (blue line). Also shown are S(Q) structure factors derived from the MD simulations for the dry albite glass (green line) and for the hydrous albite melt (purple line). The experimental S(Q) for a dry albite melt measured by Yamada et al.^25^ at 1500 °C and 5.3 GPa (red line) is shown here for comparison.

The experimental S(Q) structure factors for the dry albite glass and the hydrous albite melt are shown in Fig. 1. Also shown for comparison are the S(Q) structure factors obtained from the MD simulations made on the dry albite system and hydrous albite melt. The agreement between the modeled and experimental S(Q) for the dry albite glass is very good. Although the agreement between the modeled and experimental S(Q) for the hydrous albite melt is not quite as good as that for the dry albite glass, the modeling still gets the majority of the salient features correct. Some of the inconsistency is in part due to issues in the experimental data processing, as is particularly evident in the 14–16 Å^−1^ Q range. The first-sharp diffraction peak (FSDP) occurs at 1.76 ± 0.01 Å^−1^ in the S(Q) measured from the hydrous albite melt, which is slightly lower than the FSDP position of 1.87 ± 0.005 Å^−1^ found in the S(Q) of the dry albite glass. This is suggestive of a slightly lower density structure of the hydrous albite melt in comparison to that of the dry albite glass. As shown below, the lower position of the FSDP is consistent with the increase in the ring size occurring in the network structure of the hydrous albite melt^24^. The S(Q) measured from a dry albite melt at 1500 °C and 5.3 GPa by Yamada et al.^25^, having an FSDP at 2.059 Å^−1^, is also shown for comparison in Fig. 1. The shift of the FSDP to a higher position in Q is attributed by the authors to an overall densification of the aluminous silicate network due to considerable pressure exerted on the albite melt. The general similarity of the second diffraction peak (SDP) at about 5 Å^−1^ in the S(Q) measured both from the hydrous albite melt and the dry albite glass indicates similar topological short range order in both^26^. However, the overall SDP shape is somewhat differentiated in the S(Q) of the hydrous albite melt when compared to that of the dry albite glass, which may be due to incorporation of OH and H_2_O in the structure of the former and not the latter.

**Figure 2:**
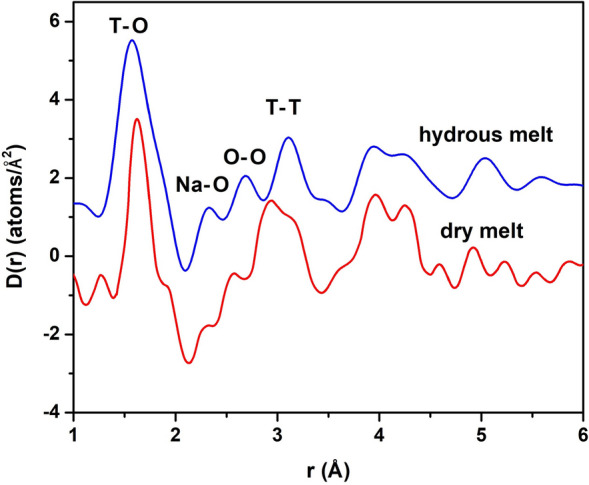
The total differential distribution function D(r) for the hydrous albite melt at 800 °C and approximately 0.3 GPa (blue line) and for the dry albite melt measured by Yamada et al.^25^ at 1500 °C and 5.3 GPa (red line).

In Fig. 2 is shown the total radial distribution function D(r) obtained for the hydrous albite melt and that of the dry albite melt measured by Yamada et al.^25^ at 1500 °C and 5.3 GPa. Due to the temperature limitations of our sample cell, we were unable to make in situ high-energy X-ray diffraction measurements of an albite melt under comparable conditions. The T–O peak is noticeably broader in the D(r) of the hydrous albite melt than that of the dry albite melt, potentially due hydroxylation in the former. The O–O and T–T peaks occur at longer r distances for the hydrous albite melt than that of the dry albite melt. This is consistent with the increase in the amount of non-bridging oxygens (NBOs) and in the overall size of the rings in the network structure of the hydrous melt. In addition, whereas the overall T–T peak appears to be a convolution of two principal components for the dry albite melt, the same peak is unimodal for the hydrous albite melt. This difference may be in part due to the much higher P–T conditions, particularly the pressure, under which the dry melt (5.3 GPa) was measured in comparison to those for the hydrous albite melt (0.3 GPa).

**Figure 3:**
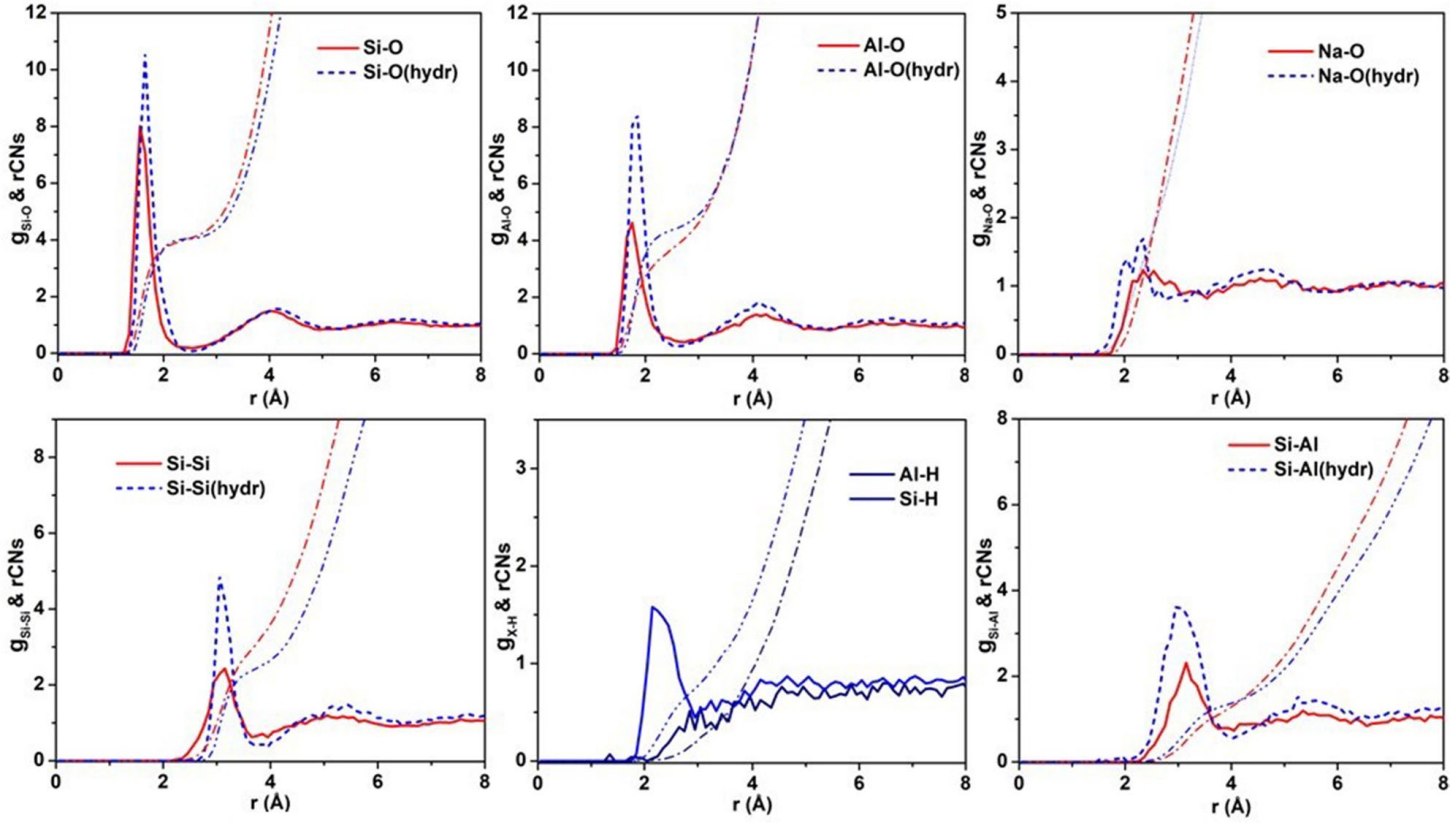
Select g_ij_(r) partial pair distribution functions and the corresponding running CNs (rCNs) for the simulated dry (solid red lines) and hydrous (dashed or solid blue lines) albite melts. The plots for the remaining g_ij_(r)‘s and running CNs are presented in Fig. S3. Note that the g_Al-O_(r) was calculated with oxygens originating from the albite structure and from the H_2_O dissolved in the melt under simulated high P–T conditions.

Next, we make an analysis of the structural results obtained from the MD simulations of the hydrous and dry albite melts. Figure 3 shows select partial pair distribution factors g_ij_(r)’s and corresponding running coordination numbers (rCNs) for the hydrous and dry albite melts. The bond lengths, near neighbor and next-near neighbor distances were determined from the location of the first peak in the g_ij_(r) partial pair distribution functions. We have compared the T–O bond lengths, O–O, Si–Si and other relevant distances obtained from our g_ij_(r)’s to those determined for a hydrous Na-silicate melt from ab initio MD simulations by Poehlmann et al. and found these to be generally in excellent agreement (see Table 1)^27^. The only notable differences are the slightly longer Si–Si distance and slightly shorter Si–H distance from our results compared to those of Poehlmann et al. The Si–O and Al–O bond lengths, are found to be slightly elongated in the hydrous albite melt (1.65 and 1.81 ± 0.02 Å) compared to those in the dry albite melt (1.59 and 1.74 ± 0.02 Å), respectively. This is consistent with the density decrease in the hydrous albite melt structure, in comparison to that of the dry albite melt, due to siloxane bond breaking and hydroxylation.

**Table 1:** A comparison of interatomic distances in the hydrous albite melt estimated from calculated g_ij_(r)’s.

A_i_–A_j_ distance	Our results (Å)	Poehlmann et al. results (Å)
Si–O	1.65	1.65
Na–O	2.31	2.32
Si–Si	3.07	3.03
Si–H	2.26	2.3
H–O	0.98	0.99
O–O	2.71	2.70
H–H	1.54	–

As shown in Fig. 3, whereas each Si is surrounded by four O atoms as measured from the inflection point in the rCN, whether in the dry or hydrous albite melt, each Al is similarly surrounded by about four O atoms in the dry melt but this increases by nearly ten percent more O atoms in the hydrous melt (~ 4.4). This is suggestive of some fraction of fivefold O-coordinated Al in the depolymerized hydrous albite melt. The Na–O bond length, as determined from the first peak of the g_Na-O_ pair distribution function, is reduced in the simulated hydrous albite melt (2.31 ± 0.03 Å) compared to the dry albite melt (2.46 ± 0.05 Å), the former value being in good agreement with the Na–O bond length in the simulated hydrous Na-silicate melt as determined by Poehlmann et al.^27^. The O–H bond length and H–H distance shown in Table 1, as determined from the first peak of the g_H-O_(r) and g_H-H_(r) partial pair distribution functions (see Fig. S3), are consistent with known values in Si–O–H bond formation and in H_2_O, respectively. Our g_Si-H_(r) and g_Al-H_(r) partial pair distribution functions and the respective rCN results shown in Fig. 3 indicate that whereas there is a correlation between Si–H, there is very little or no correlation between Al–H in the hydrous albite melt. This is suggestive of predominant hydroxylation occurring at the siloxane bond sites in a hydrous albite melt.

We next examine the Si–Si, Si–Al, Al–Al, and O–O correlations, which reflect the intermediate structure of the hydrous vs dry albite melts. Based on the g_Si-Si_(r) and g_Al-Al_(r) partial pair distribution functions, whereas the Si–Si distance is unchanged (3.09 ± 0.04 Å), the Al–Al distance is substantially reduced in the hydrous albite melt in comparison to that of the dry melt, being 3.08 ± 0.10 Å in the former and 3.25 ± 0.07 Å in the latter, respectively. It should be noted that the g_Al-Al_(r) partial pair distribution function exhibits an overall peak shape suggestive of a bimodal distribution where the lower r-side contribution is predominant in the hydrous melt and a minor contribution in the dry albite melt. The location of the first peak of the g_Si-Al_(r) partial pair distribution function from the MD simulations shows that the Si–Al distance is reduced to a value of 3.07 ± 0.05 Å in the hydrous albite melt relative to the value of 3.15 ± 0.05 Å in the dry melt. In addition, the Si–Si running CN values for the albite hydrous melt are reduced (~ 2.49) relative to that of the dry melt (~ 3.21). The position derived from the first peak of the g_O-O_(r) partial pair distribution function shows that the O–O distance in the hydrous albite melt is increased to 2.73 ± 0.05 Å from a value of 2.64 ± 0.05 Å in the dry melt. These results are consistent with a reduction in the density and depolymerization of the network structure of the hydrous albite melt.

**Figure 4:**
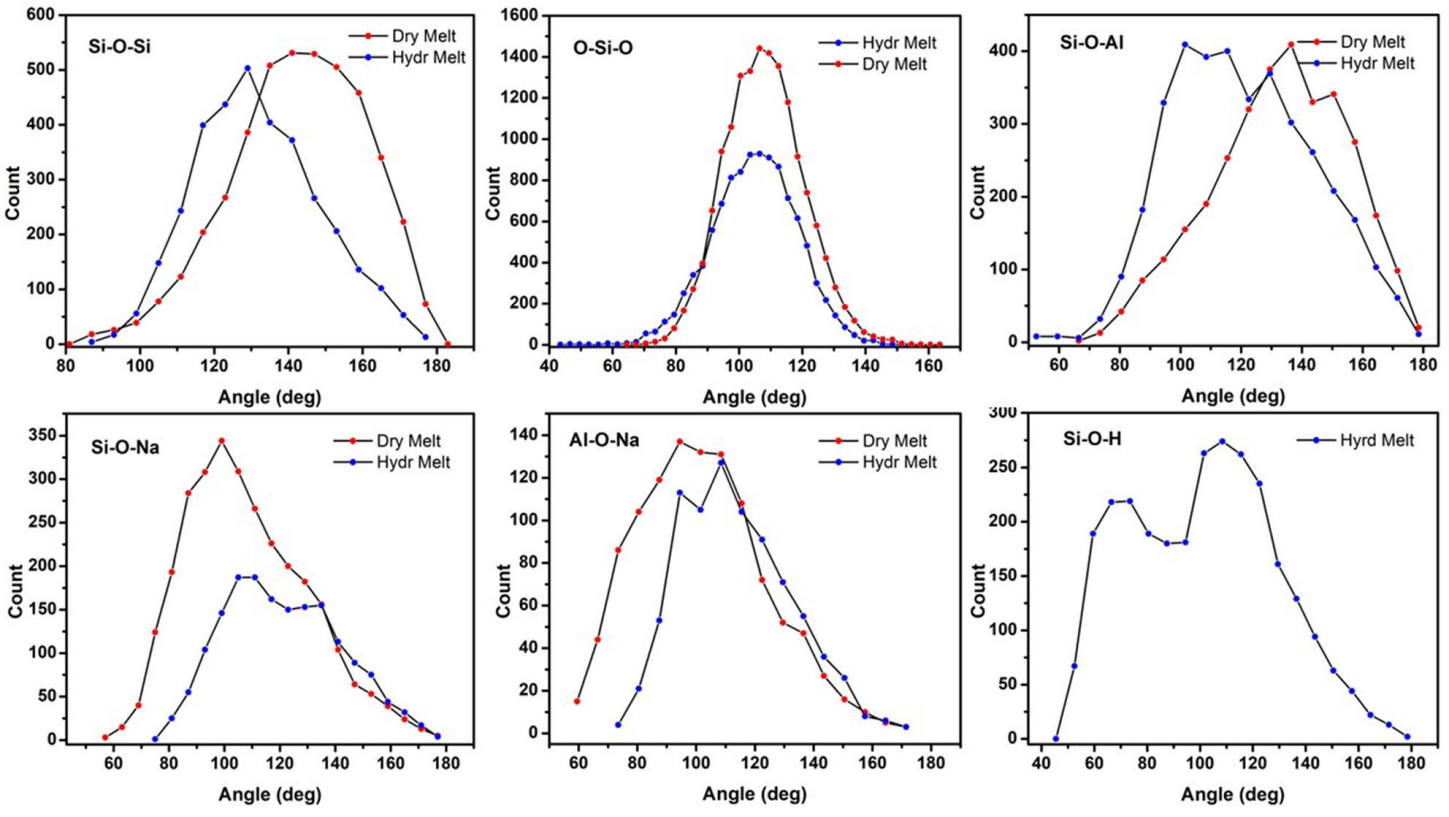
The bond angle distributions calculated from the snapshots taken from the simulations of the dry and hydrous albite melts.

Figure 4 shows the bond angle distributions in the simulated hydrous albite melt in comparison to those of the dry albite melt. The Si–O–Si bond angle distribution data show a maximum at about 145° for the dry melt whereas the same is decreased to about 130° for the hydrous melt. The distribution for the dry melt shows a small tail at about 90°, which is consistent with two SiO_4_ units joined along the edge, whereas this is absent for the hydrous melt. The Si–O–Al bond angle distribution data shows a similar trend with a maximum at about 137° for the dry melt being shifted to about 115° for the hydrous melt. In addition, the distribution of the Si–O–Al bond angles is broader for the hydrous albite melt than for the dry albite melt indicating greater structural disorder in the former. The O–Si–O bond angle distribution maximum is consistent for both the dry and hydrous melt at about 108°, which is very close to the ideal tetrahedral angle of 109°. There is slight redistribution from larger to smaller angles for the hydrous melt, in comparison to the bond angles for the dry melt.

The Si–O–Na bond angle distribution shows a maximum at about 100° for the dry melt. Conversely, the same bond angle distribution appears to have a bimodal character for the hydrous melt with lower-angle maximum at about 110° and the higher-angle maximum at about 135°. The Al–O–Na bond angle distribution shows a similar trend: The maximum in the bond angle distribution is ~ 100° for the dry melt and at about 110° for the hydrous albite melt. The Si–O–H bond angle distribution has a bimodal character with one maximum at approximately 70° and the other at about 110°. We conjecture that the higher-angle maximum occurs as a result of depolymerization and hydroxylation of the albite melt network structure whereas the lower-angle is from H_2_O molecule filling of voids in the same structure.

**Figure 5:**
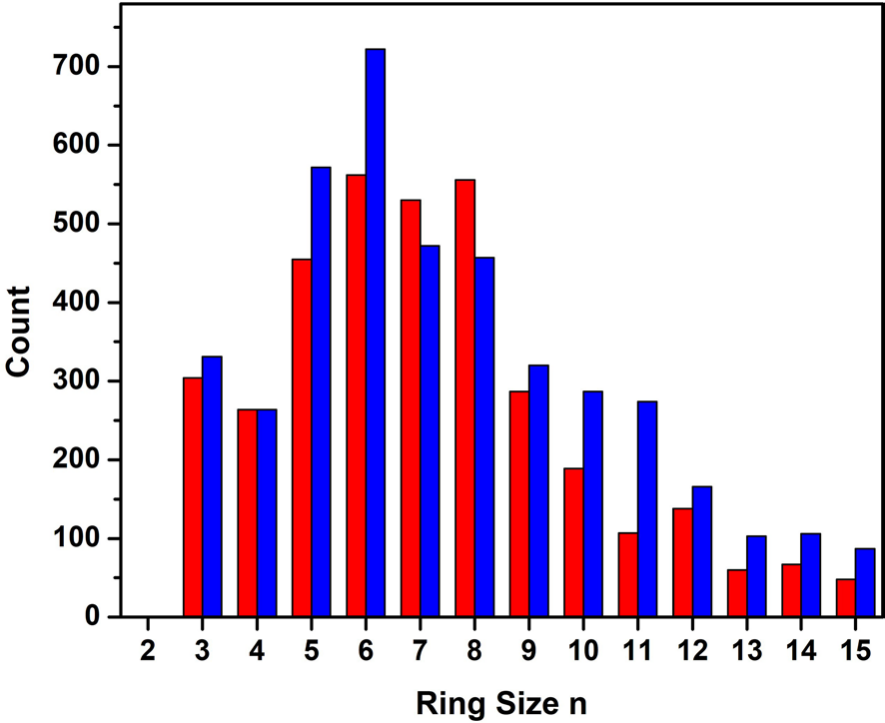
The number of King’s rings as a function of the number of nodes obtained from the MD simulations of the hydrous albite melt (blue) and dry albite melt (red).

Figure 5 shows a comparison of the number of King’s rings calculated from snapshots of the MD simulation of the hydrous albite melt and that of the dry albite melt. For this study, a King’s ring is the shortest connected path between two nearest neighbor O atoms in the melt network that loops back to a given T atom (i.e., node). In order to assess the extent of polymerization and intermediate structure, the King’s rings were calculated by considering only the nodes of the tetrahedral network forming species Si and Al and accounting for only homopolar bonds. Whereas the majority of the rings in the dry albite melt contain 6–8 nodes, most of the rings in the hydrous albite melt contain only 6 nodes. Clearly, there is a shift to an increase in smaller node rings (n ≤ 6) at the expense of larger node rings (7 and 8) in the hydrous vs the dry albite melt. In addition, there is an increase in the number of rings having greater than 9 nodes in the hydrous vs the dry albite melt. Overall, this is indicative of a shift from a predominantly unimodal ring distribution in the dry albite melt to quasi-bimodal peaking roughly at 6 and 11 nodes for the hydrous albite melt. This result is attributed to modification of the albite melt network structure, caused by incorporated water, upon breaking of Si–BO bonds and subsequent Si–OH bond formation. The increase in the higher node rings has been correlated with an increase in the NBO’s, which had been conjectured to prevent ring closure in the network NaO_2_⋅xAl_2_O_3_(3-x)⋅3SiO_2_ glasses^28^. Furthermore, such a shift toward higher node rings is consistent with a more open network structure and a lower position of the FSDP in the measured S(Q) of the hydrous albite melt.

The modifications of the intermediate structure as evidenced from our results, namely the reduced density and the variations in the atom–atom distances, bond angle distributions, running CN values, and the size of the King’s rings, are consistent with an extensively depolymerized network structure of the albite melt upon dissolution of H_2_O. Similar results for depolymerization were found from recent ab initio MD simulations by Bajgain et al.^29^, and from classical MD simulations incorporating unique force fields by Dufils et al.^30^, of hydrous aluminosilicate melts. Whereas previous experimental studies have inferred how the intermediate structure of silicate melts is modified upon water dissolution, either from measurements of quenched hydrous glasses or spectroscopic measurements of hydrous melts at high P–T conditions, ours is a first direct structural study to provide such data.

The Burnham^18^ model is predicated upon dissolution of H_2_O in a silicate melt and breakage of the T–O bonds within the network structure, resulting in the formation of T–OH^–^ groups, i.e., hydroxylation. At up to 50 mol% H_2_O content, this chemical reaction was described by Burnham^18^ as follows:

$${\text{NaAlSi}}_{{3}} {\text{O}}_{{8}} + {\text{ H}}_{{2}} {\text{O }} \to {\text{ NaAlSi}}_{{3}} {\text{O}}_{{7}} \left( {{\text{OH}}^{-} } \right)_{{2}}$$

At H_2_O content beyond 50 mol%, the dissolution mechanism in the Burnham model involves further T–O bond breakage and additional T–OH^–^ group formation along with residual number of OH^–^ ions within the melt. However, Stolper reported from IR spectroscopic studies presence of both OH^–^ and H_2_O within hydrous silicate glasses, including albite^5^. Although the IR measurement of OH^–^ and H_2_O was assumed to represent the equilibrium speciation in silicate melts, Dingwell and Webb showed that the speciation values are quench-dependent in hydrous glasses^14^. Inspection of snapshots taken from our MD simulations of the hydrous albite melt shows coexistence of OH^–^ and H_2_O within the network structure. The fact that these simulations were used successfully to model the S(Q) determined from the XRD measurements of the hydrous albite melt highly suggests that both OH^–^ and H_2_O persist within the melt structure at 800 °C and 300 MPa. This finding provides additional merit to the need for more sophisticated models of the dissolution mechanism of water in silicate melts.

Mysen et al. reported on results from Raman spectroscopic studies purportedly providing evidence for Al^3+^ dissociation from the tetrahedral network of hydrous silicate melts upon dissolution of H_2_O and ensuing hydroxylation reactions^31^. Our results from MD simulations show that the running CN for Al–O correlation (Fig. 3) is slightly greater than 4 in the hydrous albite melt. In addition, the T–O peak of the D(r) obtained from the XRD measurements of the albite–water system at 800 °C and 300 MPa (see Fig. 2) is found to be of comparable relative intensity as the same peak in the D(r) for the dry albite melt measured at 1500 °C and 5.3 GPa by Yamada et al.^25^, suggesting the same overall tetrahedral T–O environment in the hydrous and dry albite melts. This evidence agrees with the results from X-ray diffraction measurements of hydrous albite glasses by Okuno et al.^23^ but contradicts the conclusions made by Mysen et al.^31^.

Our results clearly show that the Na^+^ ion plays an important role in the modification of the intermediate structure upon water dissolution in the albite melt at high P–T conditions. Based on our MD simulation results, the Na–O distance is reduced by about 6% in the hydrous albite melt compared to that of the dry albite melt. The Na–O distance (2.31 ± 0.03 Å) is in excellent agreement with the location of the Na–O peak of the D(r) determined from the X-ray diffraction measurements of the hydrous albite melt (see Fig. 2). We note that a similar feature occurs in about the same location in the D(r) of the dry albite melt obtained by Yamada et al.^25^ However, we attribute this to the reduced density at much greater P–T conditions (1500 °C and 5.3 GPa) under which the dry melt was measured. The progressive densification of anhydrous aluminosilicate liquids with increasing pressure has been well documented^32^. Cody et al. reported indirect evidence for NaOH complexes in quenched hydrous Na-silicate glasses from NMR measurements^33^. This was attributed to a reaction between an H_2_O and Na^+^ bonded to a nonbridging oxygen (NBO) to yield a bridging oxygen (BO) and a NaOH complex. Thus, formation of NaOH in hydrous Na-silicate melts should lead to increased polymerization. However, a more recent ^23^Na NMR study by Simakin et al. discounts any measurable presence of NaOH complexes in hydrous Na-silicate melts or glasses^34^. Our MD simulations also do not show any presence of NaOH complex formation from direct examination of numerous snapshots of the modeled hydrous albite melt. Given that our MD simulations are able to reproduce the Na–O structural features of our XRD measurements made on the albite-H_2_O system, we infer that NaOH complexes do not occur in hydrous albite melts. Furthermore, the Na–NBO (NBO: nonbridging oxygen) bond has been shown to be shorter than the Na–BO (BO: bridging oxygen) bond in silicate glasses^35,36^. The NBO/T ratio is expected to increase in proportion to the BO/T ratio upon depolymerization of a silicate melt. Thus, the reduction in the Na–O bond length in the simulated hydrous albite melt in comparison to the same in the dry albite melt is attributed to an increased degree of Na–NBO bonding, in relation to Na–BO bonding, in the albite melt upon dissolution of water (see Fig. S4).

The fact that breaking of the siloxane bond and Si–OH bond formation occurs upon dissolution of water in silicate melts is well established^10,12,13^. However, whether a similar mechanism is responsible for the breaking of the Al–BO bond and subsequent Al–OH bond formation has been a subject of debate. Evidence for the absence of Al–OH was reported by Kohn et al. from NMR measurements of hydrous aluminosilicate glasses^37^. The results from XRD measurements of hydrous albite glasses by Okuno et al. are consistent with this result^23^. Conversely, Sykes and Kubicki^38^ proposed a water dissolution model in aluminosilicate melts and suggested that a Raman band occurring near 900 cm^−1^ is evidence of Al–OH stretching mode based on measurements by Mysen and Virgo^39^. However, Sharma et al. showed from Raman measurements of hydrous albite glass that the band occurring near 900 cm^−1^ was instead due to the anti-symmetric stretching modes involving network BO’s (i.e., T–O–T linkages)^40^. From our calculated g_Si-H_(r) obtained from the MD simulations of the hydrous albite melt, we obtain an Si–H distance of 2.26 ± 0.03 Å, which is consistent with Si–OH bond formation. Conversely, from the calculated g_Al-H_(r) partial pair distribution function we find no evidence for Al-H correlation and thus, conclude that our MD simulations show negligible Al–OH bond formation in the hydrous albite melt (see Fig. S4). This result is consistent with ab initio calculations made on cluster models used to represent water dissolution in albite by Liu et al.^41^ As an alternate to Al–OH bond formation, Kohn et al.^42^ have proposed that H^+^ exchanges for Na^+^ in the network structure and NaOH formation occurs in hydrous albite melts.

Our results clearly show that the albite melt undergoes appreciable depolymerization and a reduction in overall density upon dissolution of water at pressures and temperatures that are consistent with crustal conditions. Based primarily on experimental evidence obtained from hydrous albite glasses, Kohn has suggested that hydrous albite melts exhibit a relatively low degree of depolymerization and thus, an enhanced stability of the associated activated complexes provides the driving mechanism causing the reduction of viscosity under high P–T conditions^13^. However, our results suggest that the extent of depolymerization, based on the substantial modifications of the intermediate structure of the hydrous albite melt discussed above, is more significant than postulated by Kohn. This may be due to several factors, including the inherent structural differences between a hydrous glass and melt (i.e., saturation vs exsolution) and that much of the previous evidence for the structure of hydrous glasses was obtained indirectly from spectroscopic measurements (NMR, IR, Raman, etc.). Furthermore, as discussed above, we do not see evidence from our results for activated NaOH complexes in the hydrous albite melt under high P–T conditions thus throwing doubt on the model whereby the enhanced stability of such complexes leads to a reduction of viscosity of albitic melts under high P–T conditions. We speculate that the modifications to the intermediate structure, namely the expansion in T–O bond lengths, greater extent of Na–NBO and Si–OH bonding, and potentially other related factors cause the reduction of viscosity of hydrous granitic melts at high P–T conditions.

Clearly, our results on the modifications of the intermediate structure of albite upon incorporation of water at high P–T conditions have to be incorporated in the development of refined models of water dissolution in Na-bearing aluminosilicate melts. These results include Si–BO bond breaking upon reaction with water, leading to pronounced depolymerization with subsequent Si–OH bond formation and negligible Al–OH formation. Furthermore, we do not find any evidence for dissociation of Al^3+^ from the network structure as reported from previous studies^31^, upon siloxane bond breaking and depolymerization reactions in the hydrous albite melt. Our results show that the Na^+^ ion plays an important role in the modification of the network structure of a silicate melt upon water dissolution. Rather than dissociating from the network structure with depolymerization reactions leading to formation of NaOH complexes, the Na^+^ ion persists as a structure modifier but shifts from Na-BO bonding to a greater extent of Na-NBO bonding in accordance with more pervasive depolymerization of the network. Our MD simulation results show that the T–O bond lengths undergo an expansion of about 6% in the hydrous albite melt compared to those of the dry melt, under high P–T conditions. Nevertheless, our work underscores the need for further in situ X-ray diffraction structural studies of albite and other silicates in contact with water at high P–T conditions. Notably, this should also ideally include neutron diffraction (ND) studies on the same systems under high P–T conditions as these will provide important evidence on the role of OH^–^, H_2_O and associated species. Progress on ND studies however is contingent upon development of a suitably large sample-volume hydrothermal pressure cell.

## Methods

The in situ high-energy XRD measurements were made using our modified hydrothermal diamond anvil cell (HDAC)^43^. The sample volume is defined by two opposing 1/8 carat diamond anvils and a cylindrical recess milled in the center of the culet face of one of the anvils. In this way, the sample consisting of an albite glass chip and water only makes contact with diamond. The culet face of each diamond anvil measures 1 mm in diameter and the recess measures 300 μm in diameter by 40 μm in depth. The temperature of the cell is controlled using a Linkam  TS1500 temperature controller that is coupled with resistive heaters wound around silicon nitride seats that support the diamond anvils. Temperature measurement is made using Pt/Rh thermocouples that are in contact with each of the anvils and read with an accuracy of ± 0.5 °C. The sample pressure of the sample was determined by use of the quartz α–β transition to calibrate several iso-T_H_ curves, where T_H_ is the vapor–liquid homogenization temperature, in P–T space: this method has been described in more detail in Anderson et al.^43^.

In situ high-energy X-ray diffraction measurements of the hydrous albite melt were made at 800 °C and 300 MPa at sector 6-ID, at the Advanced Photon Source (APS). At these P–T conditions, the water-saturated albite melt being analyzed contains approximately 8 wt.% H_2_O^44^. Beamline 6-ID-D uses a bent-Laue Si(311) monochromator to provide a high-energy incident X-ray beam. The X-ray diffraction measurements were made at an X-ray energy of 100 keV (λ = 0.123628 Å). The incident X-ray beam, which was collimated to a spot size of 200 × 70 μm to correspond with the size of the sample in the HDAC, was positioned at 90° to the compression axis of the cell. As was shown in our previous work, this X-ray beam-cell geometry offers distinct advantages for probing the sample in providing for a short X-ray path length (∼ 700 μm) through a single diamond anvil^43^. The short path length substantially reduces the Compton scattering contribution to the background in the X-ray diffraction signal. Our HDAC design allows for collection of diffraction patterns at up 30° in solid angle, which in turn enables an integrated signal intensity from 0.5 to 30 Å^−1^ in Q-range^43^. The X-ray diffraction patterns measured from the sample were imaged using an PerkinElmer Digital XRD 1621 NES area detector. The detector was positioned ~ 353 mm from the sample. The area detector exposure time was set at 2 s: The short exposure time was necessary to avoid saturation due to the Bragg peaks from the diamond anvil. A total of 60 frames were collected and averaged for each measurement to increase the signal-to-noise statistics. Each such collection was repeated for a total of 6 times for each measurement. The dark current signal was subtracted from all averaged X-ray diffraction data. Using methods described previously, the background signal was measured from the diamond anvil at a position just below the sample^43^. XRD measurements were made of a crystalline CeO_2_ crystalline standard in the HDAC, in order to make a geometrical correction to the measured intensity of the sample for proper Q-scale calibration. In addition to measurements made on the hydrous albite melt, identical X-ray diffraction measurements were made of a crushed sample of the dry albite glass in polyamide tubing.

The data analysis procedures that were used for the X-ray diffraction data measured in this study are the same as was outlined in our previous study^43^ and will only briefly be outlined here. The program Fit2D was used in the masking, integration, correction for polarization and geometric factors, and background subtraction of the image plate data^45^. A mask was used to remove Bragg peaks from the diamond and shadows from the setup in the X-ray diffraction images. The integrated intensity data was converted into intensity I(Q), where Q is the transfer momentum, using PDFGetX2 software^46^. In the process, the data are corrected for Compton and multiple scattering, oblique incidence, and sample-dependent absorption. The I(Q) data were normalized to the elemental composition of the sample. The structure factor S(Q) data were generated using the PDFGetX2 software by use of the following formula, S(Q) = I(Q)/|f(Q)|^2^. The f(Q) term is a summation of the elemental, Q-dependent atomic form factors for each sample.

A computational cell containing 3250 atoms of stoichiometric albite (NaAlSi_3_O_8_) was rendered using the OVITO visualization software^47^. All molecular dynamics (MD) simulations were carried out using the LAMMPS code^48^. Using suitable inter-atomic potentials, LAMMPS calculates the most probable incremental trajectory at each time step and for each atom in the simulation, based on energy, charge, and relative position to nearest neighbors. The Coulomb-Buckingham potentials that have been demonstrated to work well on aluminosilicate glass systems previously were used to model the inter-atomic interactions to produce an albite glass^49^. The systems was first relaxed at 300 K at zero pressure conditions. The system was then stabilized under constant number, pressure and temperature (NPT) conditions at 300 K and an isobaric pressure of 2960.8 bars for 5000 timesteps of 1.0 fs per step. The cell was subsequently heated from 300 to 5000 K over 50,000 timesteps (50 ps) at a rate of about 94 K/ps under NPT conditions. The system was then held at 5000 K for a period of 5000 timesteps and subsequently quenched to 300 K over 10,000 timesteps at a rate of 470 K/ps. The system was then allowed to relax for another 10,000 timesteps while held at 300 K. In order to achieve optimal glass properties, the system was again cycled through a melt-quench process by heating to 5000 K over 25,000 timesteps (25 ps) under NPT conditions while held at a pressure of 2960.8 bars, at 5000 K for 5000 timesteps (5 ps). The system was subsequently quenched to 300 K over 10,000 timesteps at a rate of 47 K/ps. Then it was reheated to 5000 K over 25,000 timesteps, then kept at 5000 K for another 25,000 steps and then quenched at the same rate. A Barenson thermometer was used in the simulation process.

The inherent structure energy (ISE) of the system was utilized to ensure observation of the crystalline to glass transition and to determine the scaling of the glass transition temperature T_g_^50^. In this case, the system was heated to 5500 K at about 3.0 kbar under NVT conditions and left there for 10,000 timesteps (10 ps). The temperature was then lowered to 5000 K over 5000 timesteps and kept there for another 10,000 timesteps. These steps were repeated until the temperature was lowered to and held at 1500 K, after which it was decreased in 200 K increments until 300 K was reached. The overall simulation was used to extract 10 ISE values, normalized to the average ISE value of the system at 300 K, from equal time intervals for each held temperature, to determine the T_g_ of the albite system (see Fig. S3). Following the methodology of Bouhadja et al.^50^, the T_g_ value of 2020 K for the simulated albite glass was determined from the intercept of the linear fits of the landscape influenced and glass branches of the ISE vs temperature data. The substantial difference between the experimentally measured and the T_g_ value determined from our MD simulations is in part due to the difference in quenching rates for the two methods. The difference in T_g_ values is also partially attributed to the periodic boundary conditions used in the MD simulations, effectively making an infinite solid devoid of a surface, whereas initiation of melting of solids occurs at surfaces. The final timestep from the simulation output was utilized as the dry glass for subsequent simulation modeling.

The MD simulations of the hydrous albite melt were carried out utilizing reactive force field (REAXFF) parameters first obtained by Lyngdoh et al.^51^. The REAXFF potentials are necessary to simulate the water-melt chemical reactions. The hydrous albite glass simulation cell is shown in Fig. S1. The simulation cell was constructed by arranging 216 water molecules in each of two side slabs of a central slab composed of the dry albite glass of 3250 atoms, that was obtained using the steps outlined above, using PACKMOL^52^. The cell was relaxed at 300 K using NVT conditions for 1000 timesteps with each timestep lasting for 0.25 ps. This timestep (0.25 ps) was used throughout the simulation of the hydrous albite system. The system was then heated to 5000 K over 10,000 timesteps and then held at 5000 K for 25,000 timesteps. The hydrous melt was then cooled to 4000 K over 10,000 timesteps, at a rate of 0.4 K/ps, and subsequently allowed to relax for another 10,000 timesteps at this temperature. The simulation of the hydrous melt was then switched to NPT, with the pressure held at 2960.8 bars at 4000 K for 10,000 timesteps. Finally, the hydrous albite melt was quenched to 300 K over 10,000 timesteps and allowed to relax to a hydrous glass.

The modeled structure factor S(Q)’s were obtained from the trajectories of the simulated dry albite melt and the hydrous albite melt using the TRAVIS software^53^. The partial pair distribution functions (g_ij_(r)'s), integrated coordination numbers (ICNs), and bond angle distributions were obtained from the trajectories of the simulations of the dry albite glass, dry albite melt and of the hydrous albite melt using LAMMPS. The temperature, volume, pressure, total energy, and total potential energy of the dry and hydrous albite systems, as well as the charge, energy, and position of the individual atoms were provided in the output from the simulations for further analysis. The rings calculations were carried out using the RINGS code^54^.

## Supplementary Information

Supplementary Information 1.

Supplementary Video 1.

Supplementary Video 2.

Supplementary Video 3.

Supplementary Video 4.

## Data Availability

All data generated or analysed during this study are included in this published article (and its supplementary information files).
