# Supplementary material for: Insights on the dissolution of water in an albite melt at high pressures and temperatures from a direct structural analysis
Source: Sci Rep. 2023 Mar 10;13:4012. doi: 10.1038/s41598-023-31043-7 (PMC10006179; doi:10.1038/s41598-023-31043-7)
Supplement: Supplementary file 1 — Supplementary Information 1. [file 41598_2023_31043_MOESM1_ESM.docx]

**Supplementary Information**

**Dissolution of Water in an Albite Melt at High Pressures and Temperatures: Insights from a Direct Structural Analysis.**

Robert A. Mayanovic, Alan J. Anderson, Devon Romine, Chris J. Benmore


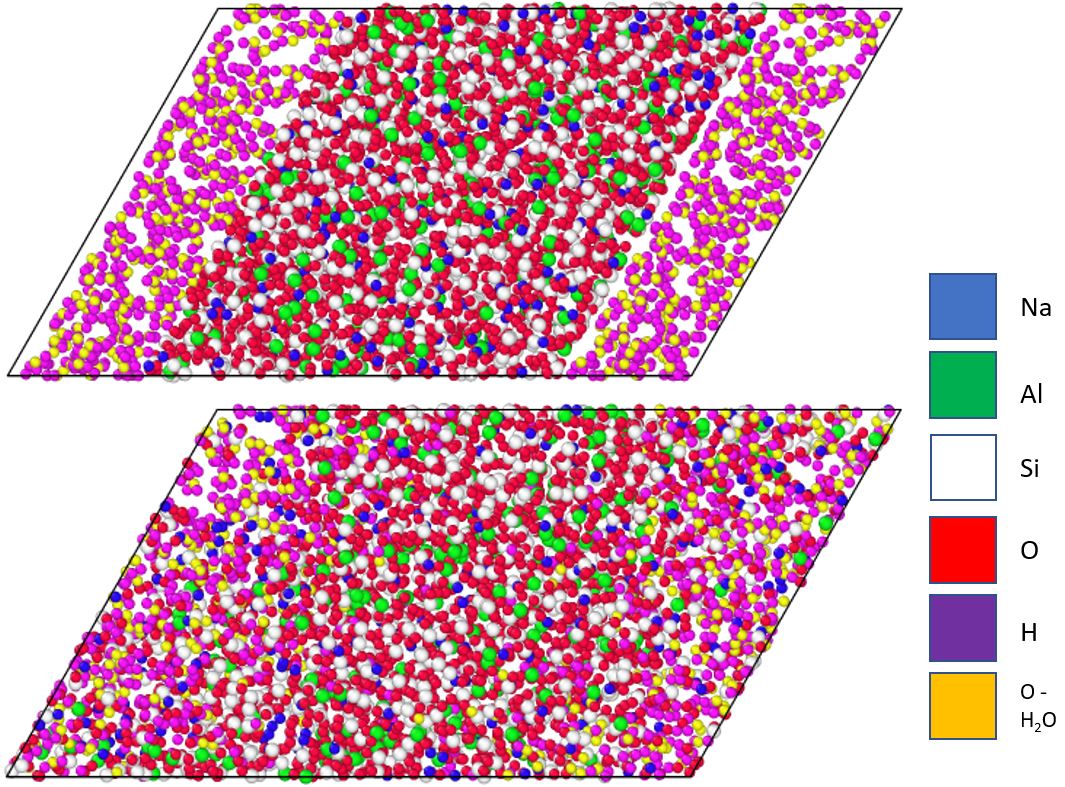


**Supplementary figure S1**. A snapshot of the MD simulation cell showing the intermediate slab constituting the albite glass and water on either side of the center slab at 300 K (top) and a snapshot of the simulated hydrous albite melt system at 4000 K (bottom). The color scheme used for individual atoms is shown to the right of the snapshots.


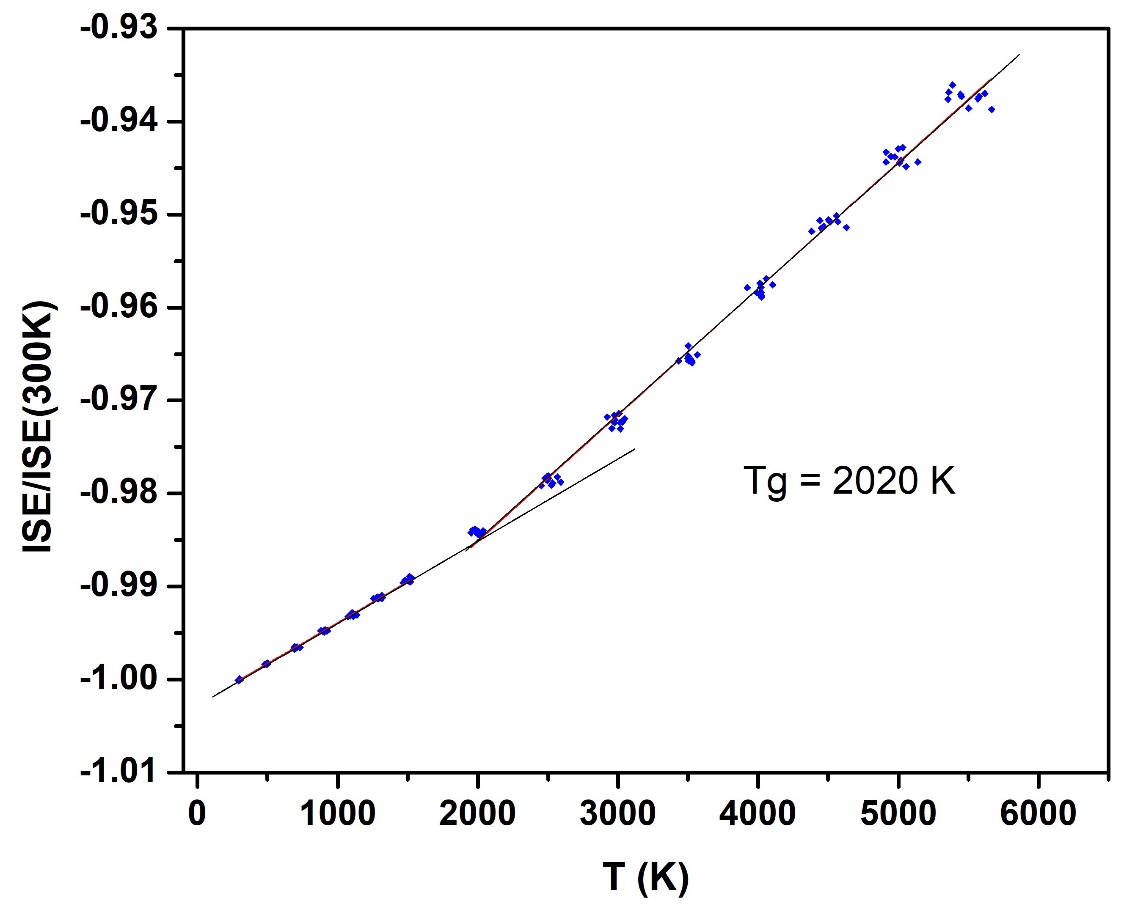


**Supplementary figure S2**. The calculated inherent structure energy (ISE) of the simulated albite glass at different temperatures (shown as points), normalized to that at 300 K. The lines are the linear fits to the calculated ISE values of the below and above the glass transition branches whereas the intercept of the two corresponds to the simulated glass transition temperature.


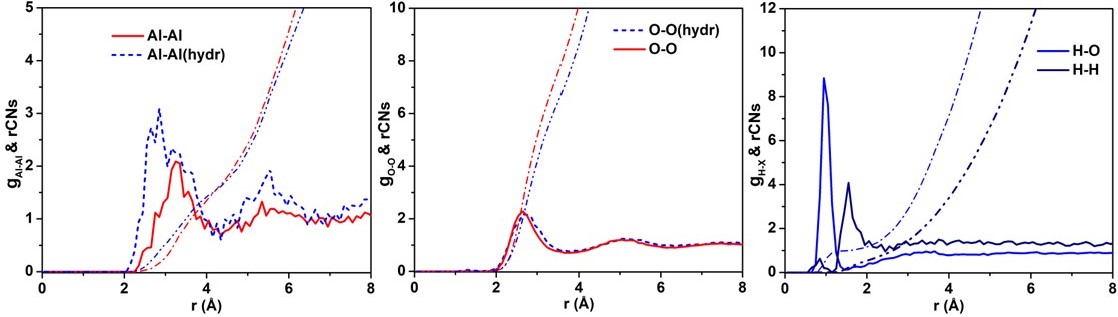


**Supplementary figure S3**. The g_Al-Al_(r) (left panel), g_O-O_(r) (middle panel), g_H-O_(r)and g_H-H_(r) (right panel) partial pair distribution functions and the corresponding rCNs for the simulated dry (solid red lines) and hydrous (dashed or solid blue lines) albite melts.

**Supplementary text 1**. Description of the calculation of S(Q) within TRAVIS software.

The TRAVIS software calculates the S(Q) by use of the partial pairwise g(r)’s and a normalization factor. The unweighted structure factor I(Q) is calculated using the following equation:

$$I\left( Q \right)= \sum_{i=1}^{N} \sum_{j=1}^{N} x_{i}x_{j}f_{i}(Q)f_{j}(Q)H_{ij}(Q)$$

where i and j are the atom types of N different types of atoms, x_i_ is the molar fraction of atom type i, f_i_(Q) is the atomic scattering factor of atom type i, and H_ij_(Q) is the partial structure factor of atoms i and j. The partial structure factor H_ij_(Q) is calculated using the equation:

$$H_{ij}\left( Q \right)=4\pi\rho_{0}\int_{0}^{r_{max}} r^{2}(g_{ij}\left( r \right)-1)\frac{sin(Qr)}{Qr}dr$$

where $\rho_{0}$ is the density, g_ij_(r) is the partial pair radial distribution function for atom types i and j, r_max_ is the maximum sampling distance in the radial distribution function, r is the radial distance, and Q is the wavevector modulus. The final S(Q) is calculated by normalization of I(Q) based on the sum of the molar fractions and atomic scattering factors of atom types i as follows:

$S\left( Q \right)= \frac{I(Q)}{\sum_{i=1}^{N} x_{i}{{(f}_{i}(q))}^{2}}$.

**Supplementary text 2**. Description of the supplemental videos.

Al-BO Environment Parts 1 and 2: A portion of a Molecular Dynamics simulation of the albite melt and water system showing the Al-bonding-oxygen environment. The color scheme for the atoms is as follows: Al (green), Si (white), Na (blue), network O (red), H (mauve), water O (yellow).

Na-NBO Environment Parts 1 and 2: A portion of a Molecular Dynamics simulation of the albite melt and water system showing the Na-nonbonding-oxygen environment. The color scheme for the atoms is as follows: Al (green), Si (white), Na (blue), network O (red), H (mauve), water O (yellow).
